# Iron Deficiency Defined by Hepcidin in Critically Ill Patients

**DOI:** 10.1186/s13054-021-03542-4

**Published:** 2021-04-12

**Authors:** Michele F. Eisenga

**Affiliations:** Department of Internal Medicine, Division of Nephrology, University Medical Center Groningen, P.O. Box 30.001, 9700 RB Groningen, The Netherlands

Lasocki et al. recently published in *Critical Care* in a controlled, randomized, single-blinded, multicenter trial that treatment of iron deficiency (ID), according to hepcidin levels, resulted in a significant reduction of the risk in mortality in critically ill patients 90 days after ICU discharge and an improved 1-year survival rate [1]. The authors defined ID by hepcidin, measured with mass spectrometry. An absolute ID was defined as a hepcidin level < 20 µg/L and a functional ID as a hepcidin level of 20 µg/L to lower than 41 µg/L. It is an interesting approach to define ID based on hepcidin levels, however, it is questionable whether this concerns the most appropriate option to determine ID.

First, in other inflammatory settings such as heart failure, it has been shown that hepcidin was not an accurate diagnostic marker for ID based on the gold standard of bone marrow iron staining [2]. When assessing bone marrow aspirates on iron stores and percentage sideroblasts (absolute vs. functional ID), hepcidin had low diagnostic accuracy, whereas a standard cut-off of transferrin saturation (TSAT) < 19.8% had excellent diagnostic accuracy. Table S3 clearly shows that patients within the intervention ID group, on which the conclusions are based by Lasocki et al., have an upper interquartile range of TSAT of 27%, questioning whether this group had ID at ICU discharge.

Second, hepcidin levels are being influenced by myriad causes [3]. The authors regard ID as the driving force behind the low hepcidin levels, even in the presence of inflammation. The latter is well-established; however, it would be of added value to exclude other potential contributing factors, and confirm that the low hepcidin levels at ICU discharge are attributable to ID. Apart from cirrhosis, no information is given about liver disease. Liver disease (e.g., chronic hepatitis C infection or auto-immune liver disease) has been shown to lower hepcidin levels already before the cirrhosis stage [4]. Similarly, alcohol consumption has been shown to lower hepcidin levels [3]. Finally, specific medications such as steroids, commonly used in the ICU setting, are not reported. Previously, we showed that 24-h urinary prednisolone levels are an important negative determinant of serum hepcidin levels in kidney transplant recipients [5]. Providing extra data about these specific aspects would further strengthen the results by Lasocki et al*.* and show that the low hepcidin levels in the intervention arm are truly attributable to ID although the TSAT levels, in part, are completely within the normal range.

## Data Availability

Not applicable.
