# Supplementary material for: Iron Deficiency Defined by Hepcidin in Critically Ill Patients
Source: Crit Care. 2021 Apr 12;25:138. doi: 10.1186/s13054-021-03542-4 (PMC8040202; doi:10.1186/s13054-021-03542-4)
Supplement: Related texts

# Reply: Authors’ response

**Authors:** Sigismond Lasocki, Samir Jaber, Thibaud Lefebvre, Sylvain Lehmann, the Hepcidane study group, Pierre Asfar, François Beloncle, Thomas Gaillard, Soizic Gergaud, Sigismond Lasocki, Maxime Leger, Emmanuel Rineau, Cyril Sargentini, Claire Geneve, Philippe Montravers, Katell Peoc’h, Herve Puy, Grégoire Mercier, Nicolas Nagot, Gregory Marin, Constance Delaby, Christophe Hirtz, Sylvain Lehmann, Gerald Chanques, Samir Jaber, Karim Asehnoune, Antoine Roquilly, Matthieu Boisson, Claire Dahyot-Fizelier, Thomas Kerforne, Olivier Mimoz, Sonia Isslame, Yoann Launey, Philippe Seguin, Mathilde Barbaz, Martine Ferrandiere

**Affiliations:** 2 Département Anesthésie Réanimation, Université d’Angers, CHU Angers, 4 rue Larrey, 49933 Angers Cedex 9, France; 3 grid.121334.6 0000 0001 2097 0141 Département Anesthésie Réanimation, Université de Montpellier, Montpellier, France; 4 grid.508487.6 0000 0004 7885 7602 Centre de Recherche Sur L’Inflammation, INSERM U1149, UFR de Médecine Bichat; Assistance Publique des Hôpitaux de Paris, APHP Nord Hôpital Universitaire Louis Mourier, - Colombes (France); Laboratoire D’Excellence GR-Ex Ou Laboratory of Excellence GR-Ex, Université de Paris, Paris, France; 5 Laboratoire de Biochimie Protéomique Clinique Et IRMB INSERM, CHU de Montpellier, Université de Montpellier, Montpellier, France

## Yes, Hepcidin is a good indicator of iron treatment in the critically ill

Michele Eisenga is right when he claims that iron deficiency (ID) diagnosis is challenging, notably in presence of inflammation. He advocates the use of a low (< 20%) transferrin saturation (TSAT) to define ID in these patients, based on a cohort of 42 patients scheduled for cardiac surgery in whom a TSAT < 20% was more predictive of ID, diagnosed on bone marrow examination, than hepcidin (measured using an ELISA method) [2]. However, these patients did not have significant inflammation (their median CRP was only 2 mg/L), contrary to ours (median CRP 68 mg/L). One should also keep in mind that TSAT is a calculated parameter, based on transferrin and serum iron measurement, and serum iron has nychthemeral variations. The timing of blood sampling is thus important. At last, in our study population, more than three-quarters of the patients had a TSAT < 20%, mainly because of inflammation [1]. Indeed, TSAT could be decreased either because of low iron stores or because of high inflammation, which prevents iron release from stores; when hepcidin is decreased in case of low iron stores and increased in presence of inflammation, so it is probably a better indicator of the balance between low stores and the impossibility to release iron (Fig. 1) [6]. Hepcidin plays a key role in iron metabolism, it prevents the release of iron by blocking the ferroportin directly. Hepcidin is thus not only “a biomarker” of ID but has a biological role. That is why we proposed hepcidin to diagnose ID in critically ill patients [7].

**Fig. 1:** Schematic representation of iron deficiency biomarker variation according to iron stores and inflammation. TSAT, Transferrin saturation. This graph represents the expected variations of TSAT (blue lines) and hepcidin (red lines), according to the level of iron stores and in absence of inflammation (left panel) or in case of inflammatory states (right panel)

Most importantly, we believe that it is more important to find a marker that predicts the response to iron therapy, rather than a marker that predicts low iron stores only. A low TSAT could be useful in heart failure patients as proposed by Grote Beverborg et al. [2], but in critically ill patients we believe that hepcidin may help improve mortality. We have previously show in a cohort of 1161 critically ill patients that a low hepcidin was associated with increased one-year mortality [8], and we now have shown in this randomized controlled trial that a treatment strategy of iron ± erythropoietin based on hepcidin quantification reduced long term mortality [1]. We will probably never have a unique definition for ID, but rather context specific markers that will help clinicians at the bedside to decide on iron treatment according to the expected outcomes. In critically ill it is probably hepcidin.
